# Siddha management of alcohol withdrawal syndrome

**DOI:** 10.6026/97320630018748

**Published:** 2022-09-30

**Authors:** E Selvasankari, V Manjari

**Affiliations:** 1 Department of Nanju Maruthuvam, National Institute of Siddha, Chennai - 47, Tamil Nadu, India

**Keywords:** Alcohol withdrawal syndrome, Mathathiyam (Indian Tamil language), Kudiverinoi (Indian Tamil language), Siddha, CIWA-Ar scale

## Abstract

Addictive use of Alcohol has been a considerable health problem, not only having impact on individual and families, but creates a lasting social burden on the society. One third of the Indian population is using alcohol in an unhealthy manner, the complications are wide and varied among which, the Alcohol withdrawal syndrome (AWS) is the dominant factor. AWS is a set of symptoms that can occur when a heavy drinker suddenly stops or significantly reduces their alcohol intake. The presentation varies from mild sleep loss or anxiety to the life threatening situation like delirium (confusion). The excessive intake of unwholesome alcohol leads to Mathathiyam (Kudiveri Noi in the Siddha - Indian Tamil language) and it degrades knowledge and health according to Siddha medicine and practise. Manifestation occur as per the aggravated/ vitiated three biological forces (Vali, Azhal, Iyyam - Indian Tamil language) leads to cause impair the quality or efficiency of life, and which may even leads to death. Hence, there is a need for AWS management at an early stage. The aim is to minimize the withdrawal symptoms which prevents complications and reduces the intense use for alcohol using the siddha system of medicines. The efficacy of Inji rasayanam (Rejuvenator), Brahmi nei (Medicated ghee), and Ammukkara chooranam (Medicated powder) for AWS is well known. Therefore, it is of interest to describe the case of a 35 years old male patient presented with AWS and treated with Siddha drugs for 48 days. The condition was assessed before and after treatment using the clinical institute withdrawal assessment for alcohol scale revised (CIWA-Ar). Data shows effective management of AWS using the Siddha medicines.

## Background:

Alcoholism is, broadly any drinking of alcohol that results in significant mental or physical health problems. Predominant diagnostic classifications are alcohol use disorder (DSM-5) or alcohol dependence (ICD-11). Excessive alcohol use can damage all organ systems, but it particularly affects the brain, heart, liver, pancreas and immune system. Alcoholism can result in mental illness, delirium tremens, wernicke-korsakoff syndrome, irregular heartbeat, an impaired immune response, liver cirrhosis and increased cancer risk. Drinking during pregnancy can result in fatal alcohol spectrum disorders. Environment and genetics are two factors in the risk of development of alcoholism. People may continue to drink partly to prevent or improve symptoms of withdrawal [1]. Alcohol withdrawal syndrome (AWS) occurs when a heavy drinker suddenly stops or significantly reduces their alcohol intake. AWS consist of symptoms and signs arising in alcohol-dependent individuals, typically within 24-48 hours of consumption of their last drink. AWS occurs intentionally in those seeking abstinence. Major symptoms of AWS are nausea and vomiting, tremor, paroxysmal sweats, anxiety, tactile disturbance, auditory disturbance, visual disturbance, headache, clouded sensorium. Abrupt cessation of Alcohol consumption by a patient with alcohol dependence may cause delirium tremens and withdrawal seizures, which may even lead to death [2]. As explained in Siddha classics, the excessive intake of unwholesome alcohol leads to Mathathiyam (Kudiveri Noi), it degrades knowledge and health. As per the text, it gives the body a certain level of pleasure and excitement with the drinks, and physical aggression. In over dose, it can cause nausea and vomiting, followed by dizziness, also show signs of sweating, pulsation, numbness of the limbs, and sometimes lead to death. Manifestation occurs as per the aggravated thathu (Biological force). In Mathathiyam, first there is an increase of firey followed by vitiation of Airy, Watery and alteration in abanan, uthanan, samanan (types of pranas-energy, vitality, power) leads to cause symptoms and signs [3]. Alcohol symptoms are occurring because of enhanced N-methyl-D-asperate (NMDA) receptor function, reduced transmission and deregulation of the dopaminergic system etc. Intervention, detoxification and rehabilitation are the three steps of management available. Siddha medicine is being practiced in South India particularly Tamil Nadu. Medicinal formulations of the Siddha system are based on herbs, metals, minerals and animal products. The proportion of the raw drugs present in the Siddha medicine is responsible for certain actions and therapeutic results.

## Methodology:

### Clinical report:

A 35 years old male patient presented in Ayothidoss Pandithar Hospital OPD- National Institute of Siddha, with symptoms and signs of tremulousness of hands, headache with decreased sleep. He started the intake of alcohol at the age of 18 till (around consume alcohol for 17 years), initially he was drink occasionally, but later frequently used to drink alcohol. He was consuming 600ml of alcohol (Liquor type) per day and 91 drinks per week. He often took more than usual amount (binge drinking). For past 7 days he stopped the consumption of alcohol then develops above mentioned withdrawal symptoms one by one.

###  Clinical observation:

Patient presented with symptoms such as tremulousness of hands, increased anxiety and agitation and decreased sleep with headache. On examination the patient was found to be anxious, the appetite was much reduced. He was of medium body built, heart rate was 78 per minute, pulse rate was 76 per minute and blood pressure was 130/80 mmHg. Amount of alcohol consumption per week was calculated it was 91 drinks. (Standard drink [1]-1 drink strands 12 g of pure alcohol, Equivalent to 5 oz of wine, 12 oz of beer, 1.5 oz of distilled spirits). So, he felt in a risk group (Men younger than the age of 65 at risk of more than 14 drinks per week).

### Management protocol:

The patient was treated with following medication for a period of 48 days (Tables 1 to 4 - see PDF). Observation was recorded before and after treatment and the data was collected.

### Assessment:

Assessment was done on the basis of changes observed at the clinical level by using Clinical Institute of Withdrawal Assessment of Alcohol revised scale [4] (CIWA-Ar scale) before and after treatment.

## Observation and results:

After 48 days of medication the progress of the patient was assessed by the presence of signs and symptoms before and after treatment based on CIWA-Ar scale. Assessment result showed in Table 5(see PDF). Patient has no tremor, no anxiety, no agitation, no headache and CIWA-Ar scale 12 before treatment became 0 (nil) after treatment.

## Discussion:

Alcohol is a major threatening contributor to global disease and a leading cause of preventable death. Although up to 50% of individuals with alcohol use disorder are present with some withdrawal symptoms after stopping the drinking, it requires medical treatment for detoxification [10]. The use of Siddha medicines in the management of Alcohol withdrawal syndrome is most needed in recent years. The drugs which treat the Alcohol related disorders mostly have the properties of sedative, anti convulsant, amnestic effect and anxiolytic effect. The selected Siddha medicines Inji rasayanam, Brahmi nei, Amukkara chooranam are useful to treat symptomatically in Alcohol withdrawal syndrome also have the following properties. The ingredients of Brahmi nei is Bacopa monnieri (Brahmi) is known for its Neurocognitive effect, act as a brain tonic to enhance learning and memory, to provide relief in anxiety or epileptic disorders [5]. Acorus calamus (Vasampu) is known for its sedative, CNS depressant, anticonvulsant, anti oxidant, hypolipidemic activity [11]. Alpinia galangal (Chitrarathai) is known for its anti oxidant, hypolipidemic, immunomodulatory activity [12]. Operculina turpethum (Sivathai root) is known for its anti inflammatory, anti oxidant and hepatoprotective actions [13]. Piper longum (Thippli) is known for its CNS depressant, anti oxidant, hepatoprotective actions [14]. Limonia acidissima (Vilankaai seed) is known for its neuroprotective, anti-hyperlipidemic activity [15]. Dried Zingiber officinale (Chukku) is known for its anti oxidant, anti inflammatory, anti emetic, anti hyperlipidemic activity [16]. Dried Phyllanthus emblica (Nellivatral) is known for its anti oxidant, analgesic, hepatoprotective actions [17]. Curcuma longa (Wild turmeric) is known for its anti oxidant, anti inflammatory and hepatoprotective actions [18]. The ingredients of Inji rasayanam is Zingiber officinalis (Inji) known for its CNS depressant, anti-emetic, anti-hyperlipidemic, stomachic actions [6]. Cuminum cyminum (Seerakam) is known for its anti inflammatory, CNS effects, hypotensive, analgesic actions [19]. The ingredients of Amukkara chooranam is Withania somnifera (Amukkara) known for its anti-depressant, anti-anxiety effect, to reduce both alcohol dependence and withdrawal thus showing anti-addictive potential [7]. Syzygium aromaticum (Lavangam) is known for its anti oxidant, anti inflammatory actions [20]. Mesua nagassarium (Siru naga poo) is a blood purifier and cardiotonic [21]. Elettaria cardamomum (Ellam) is known for its anti inflammatory, anti oxidant activity [22]. Piper nigrum (Pepper) is known for its anti oxidant, anti hypotensive, anti inflammatory activity [23]. The Patient have no signs symptoms of AWS such as tremulousness of hands, increased anxiety and agitation and decreased sleep with headache after 48 days of treatment with Inji rasayanam, Amukkara chooranam and Bhrami nei.

## Conclusions:

Data shows that a combination of Inji Rasayanam (Ginger), Amukkara Chooranam (medicated power) and Brahmi Nei (Ghee) is effective in the management of AWS. It should be noted that more such clinical data is needed in large population for further consideration.

## Author's contribution:

E. Selvasankari did the case study and literature collection. V. Manjari did the choice of treatment selection, literature review, analysis and interpretations of data, report writing and formatting.
